# How Does Consecutive Interpreting Training Influence Working Memory: A Longitudinal Study of Potential Links Between the Two

**DOI:** 10.3389/fpsyg.2018.00875

**Published:** 2018-06-05

**Authors:** Yanping Dong, Yuhua Liu, Rendong Cai

**Affiliations:** ^1^Bilingual Cognition and Development Lab, National Center for Linguistics and Applied Linguistics, Guangdong University of Foreign Studies, Guangzhou, China; ^2^College of Foreign Studies, South China Agricultural University, Guangzhou, China; ^3^School of English and Education, Guangdong University of Foreign Studies, Guangzhou, China

**Keywords:** interpreter advantage, interpreting training, consecutive interpreting, working memory, attentional control

## Abstract

With an intention to contribute to the issue of how language experience may influence working memory (WM), we focused on consecutive interpreting (CI), analyzed its potential links with WM functions and tested these links in a longitudinal experiment, trying to answer the specific question of how CI training may influence WM. Two comparable groups of Chinese learners of English received either CI or general second language (L2) training for one semester, and were tested before and after the training with the tasks of n-back (non-verbal updating), L2 listening span, and letter running span (verbal spans). CI performance was tested in the posttest. The results showed that (1) updating efficiency in both the pretest and posttest predicted CI performance, and CI training enhanced updating efficiency while general L2 training did not; (2) the relationship between verbal spans and CI performance was weaker (i.e., only pretest L2 listening span correlated with CI performance and predicted CI performance with marginal significance), and CI training did not make a unique contribution to these spans (i.e., no group differences). The results indicated an “interpreter advantage” in updating, which was probably due to that updating was more central in the CI task than WM spans. Theoretically, we believe that updating and CI are closely related because they share the same underlying mechanism, or more specifically updating and the recalling process in the CI task share the same attentional control process, a unique link between updating and the CI task. Methodological implications are discussed.

## Introduction

Working memory (WM) is considered part of the most basic executive functions that are essential to higher level cognitive processing, including language processing (e.g., Baddeley, 2003; Diamond, 2013). The relationship of the other direction, i.e., how cognitive experience such as language learning influences executive functions, is still not clear and is currently a hot topic of research (e.g., Green et al., 2014), with the issue of “bilingual advantage" as a typical example in the language domain. In our point of view, the assumption for this line of research is that the executive functions that are most essential to a certain kind of cognitive processing (e.g., bilingual processing) tend to be most influenced by corresponding cognitive experience (e.g., bilingual experience). However, the results are quite mixed, and one of the problems is that *higher-level cognitive processing (*e.g., *language processing) is complex in terms of its involvement of or* “*correspondence*" *with executive functions*, which, for convenience’s sake, the present paper refers to as *this problem of complexity*. Take bilingual processing as an example. It is believed that when compared with monolingual processing, the executive function of inhibitory control is most essential to bilingual processing because it has been found that the bilingual’s two languages are non-selectively activated, and the bilingual has to inhibit the language not needed at the moment (e.g., Green, 1998). And yet, the concept of bilingualism is complex in the sense that there are different kinds of bilingualism involving different degrees of language switching and bilingual activation (e.g., Valian, 2015; Xie and Dong, 2017), which may partly explain the inconsistent findings regarding the issue of bilingual advantage in inhibitory control (see Paap et al., 2015, for example).

The same problem of complexity exists in the issue of an “interpreter advantage” in WM. This issue has been investigated for about two decades, but no unanimous conclusion has been reached. Like bilingualism, there are different types of interpreting, but the distinction between consecutive interpreting (CI) and simultaneous interpreting (SI) is universally recognized (e.g., Liang et al., 2017) and is probably relevant to the function of WM. Few studies on the advantage issue have tried to make this distinction, and yet we believe this distinction is beneficial to reducing the influence of the complexity problem and to making further substantial progress on the interpreter advantage issue. The present study, therefore, focused on CI training (most basic and most common type of interpreting training), and tried to find out and then test its unique links with WM functions (when compared with general L2 training), hoping to answer *the research question of how CI training may influence WM*. This exploration may help identify the specific role of WM in the task of CI beyond its role in general language processing, and it may shed light on the issue of how language experience may affect WM and other executive functions.

### Interpreting Training/Experience and WM Advantage

Interpreting is one of the most difficult language tasks and its performance relies heavily on WM. The important role of WM in interpreting was not only recognized by Keiser (1965) half a century ago and in theoretical formulations such as the process models (Cowan, 1988; Mizuno, 2005), but also supported by a series of empirical studies (Padilla et al., 1995; Christoffels et al., 2003, 2006; Liu et al., 2004; Köpke and Nespoulous, 2006; Signorelli et al., 2012; Tzou et al., 2012; Timarova et al., 2014; Morales et al., 2015). However, as far as we know, the relationship between WM and interpreting is still ambiguous and controversial.

To investigate how interpreting training may influence WM (e.g., the interpreter advantage issue), previous research often compares how expert interpreters and novice interpreters (or non-interpreters) perform in WM tasks. The findings are mixed. For example, in the complex span task of listening (which is similar to the classical reading span task of WM), novice interpreters performed significantly better than both control groups in Köpke and Nespoulous (2006), but there were no group differences among professional interpreters, advanced and beginning student interpreters in Liu et al. (2004). With a comprehensive review, Dong and Cai (2015) found that the lack of consistent evidence for an interpreter advantage in WM may be attributed to several weaknesses in some previous studies, such as insufficient sample size, lack of participant control group, lack of control for factors such as age and language proficiency. Take the first factor as an example. The participant sample size was rather small in some of the studies (e.g., 10 in Padilla et al., 1995; 11 in Liu et al., 2004; 12 in Chincotta and Underwood, 1998; less than 13 in Signorelli et al., 2012), which may lead to insufficient statistical power. Brysbaert and Stevens (2018) recommended that a properly powered reaction time experiment with repeated measures has at least 1600 observations for each condition, e.g., 40 participants and 40 stimuli for each condition^1^. Apart from these weaknesses, another limitation is the fact that most previous studies have adopted a cross-sectional design. The presence of some weaknesses such as age is inherent in a cross-sectional design study because professional interpreters are generally older than novice or student interpreters. Also, a cross-sectional design cannot clarify the causation of an interpreter WM advantage. The possibility is that some personal traits, such as good WM skills, may have led the interpreter to select that particular career. In a word, more research is needed to clarify the relationship between WM and the task of interpreting or the experience of interpreting training.

### The Present Research

Previous research has made invaluable contributions, and to push the research frontier forward, the present research adopted two main measures when trying to answer the question of how CI training influences WM functions: (1) focusing on CI training and comparing it with general L2 learning experience (as control); (2) using a longitudinal design with a sufficient sample size that was well controlled in relevant background characteristics (e.g., age, intelligence, social economic status, and language learning history). These measures were intended to ensure that any WM differences between the two groups after the treatments could be attributed to the differences between the treatments.

**FIGURE 1:**
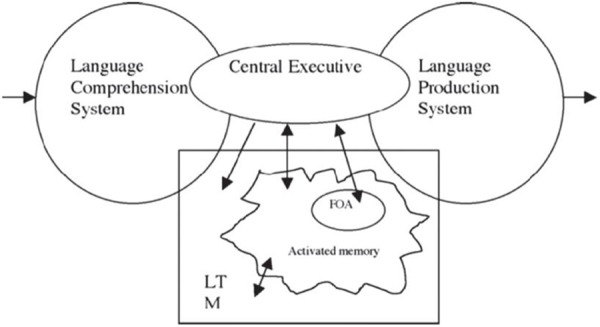
The process model of working memory (WM) and interpreting by Mizuno (2005, p. 744).

Theoretically, the embedded-processes model proposed by Cowan (1988, 1995) is most relevant to the present study. The critical idea is that human memory is a single storage system composed of elements at various levels of activation. This system can be conceived as long-term memory (LTM), in which some elements are above the threshold of activation. These activated elements, thought to be in short-term memory (STM), are outside of conscious awareness but nevertheless affect online processing such as semantic priming. Some elements in STM fall into *the focus of attention* (*FOA*) and are in a hyper-activated state, and therefore have to be maintained or manipulated with conscious effort. According to Cowan (1995, p. 100), “…WM is based on that activated information along with central executive processes,” i.e., WM is composed of the FOA and the central executive. Based on Cowan’s (1988) model, Mizuno (2005) proposed his enlarged embedded-processes model for interpreting, adding the two processes of language comprehension and production at the two sides of the original model, and emphasizing the interaction between the memory system and the language system during the process of interpreting (see **Figure 1**).

This conceptualization of WM from the perspective of embedded processes explains well not only the dynamics of the memory system, but also the difference between CI and general L2 training in terms of WM. In a CI task, the interpreter listens to a stretch of source language input, and then recalls as accurately as possible in another language what has been conveyed in the input. In general L2 training, the learner comprehends and produces but does not have to recall the messages heard. In other words, although both the consecutive interpreter and the general L2 learner have to focus on a segment of message they are listening to or they are producing, the consecutive interpreter has to recall from the beginning of a stretch of input. Using the terms in Cowan’s (1988) model, for the interpreter to recall, the Central Executive turns attention to (i.e., reactivate) the stretch of input which has already passed FOA, and the content in FOA is being updated. Although the FOA has to be constantly updated for both the interpreter and the general L2 learner to perform well in any genuine language task, interpreting or recalling as accurately as possible in another language is certainly more demanding in terms of accuracy and response time (RT) when compared with the general L2 tasks of listening, reading, speaking, and writing. In a general L2 conversation, for example, listening comprehension is important, but the conversation partner could ask for clarification if he or she does not get the message, and it is often good enough to get the gist. On the other hand, the situation is apparently more demanding for the interpreter. *We thus hypothesized that the updating function of WM was closely related to CI performance, and CI training could help enhance updating*. In addition, interpreters may process the source language input in a parallel way, i.e., their processing of the source language may be influenced by the target language (e.g., Dong and Lin, 2013), suggesting that interpreters may have a more efficient way processing the source language so that they can recall details better and perform better in tasks of verbal WM spans. Furthermore, Ecker et al. (2010) found that WM capacity was a strong predictor of WM updating, suggesting that WM spans and WM updating are closely related. O*ur second hypothesis was thus that verbal WM spans were related to CI performance, and CI training could help enhance verbal WM spans*.

To test the two hypotheses, the present study employed three WM tasks: a visuo-spatial n-back task, a L2 listening span task, and a letter running span task. Although we were fully aware that no task is pure (e.g., Valian, 2015), and the connection between a WM task and the function that the task is supposed to measure may be controversial, we tried our best to overcome potential limitations and be as specific and as accurate as possible while following most studies in the literature. *The visuo-spatial n-back task* asks participants to identify whether the current square on the computer screen is in the same location with the square presented n trials back. “The n-back task is often assumed to measure updating, with subjects actively updating the current contents of a limited portion of temporary memory” (Redick and Lindsey, 2013, p. 1111). Apparently, the n-back task matches well with the recall task in CI, although the former seems simpler and is a non-linguistic task. Using different versions of the n-back task (e.g., visuo-spatial or letter), three previous studies (Timarova et al., 2014; Morales et al., 2015; Dong and Liu, 2016) have found some relationship between interpreting training and updating ability. *The listening span task* is a complex verbal span task^2^ that requires participants to process aurally presented sentences and remember the last word of each sentence. This task has been used in the literature to test interpreters’ advantage, but the findings were mixed (Liu et al., 2004; Köpke and Nespoulous, 2006). As for *the letter running task*, we do not yet find any research using this task to explore the issue of WM in interpreting. The task requests participants to recall the last *n* letters from a series of presentation of *m + n* items, and it is believed that a fast version of the task (fast presentation of the letters) measures “the capacity of the FOA” (Lilienthal et al., 2012, p. 135) or scope of attention (Bunting et al., 2006), including attention control, its scope, and effortful retrieval (Broadway and Engle, 2010). It seems that consecutive interpreters have to do the same, controlling their attention and keeping their focus (which could be large in scope and effortful in retrieval). The latter two tasks were intended to test the second hypothesis, with the letter running task not so verbal as the listening span task.

It seems that WM tasks are rarely independent of each other, and as to empirical evidence for how exactly the three WM measures (n-back, listening, and letter running span tasks) relate to each other, there are only a few relevant findings. Briefly, Redick and Lindsey (2013) found that n-back task performance weakly correlated with complex spans (reading span), and Broadway and Engle (2010) found that the running span significantly correlated with complex spans (reading or operation spans). The relationship between n-back task performance and the running span has been discussed in the difference between active and passive input processing in the running span task. If a person processes input actively, he prepares responses (probably by rehearsing and grouping targets) in advance of a trial in a test; if a person processes input passively, he waits to prepare responses until the time of the trial, i.e., when the input presentation has been completed. It is argued that active input processing in running memory span reflects WM updating in the form of rehearsal and grouping (e.g., Morris and Jones, 1990; Conway et al., 2005; Friedman et al., 2006). On the other hand, passive input processing measures the number of items extracted from sensory memory into the FOA (Cowan et al., 2005), reflecting the size of FOA. Empirical evidence found that ONLY a slow presentation of the letters (e.g., 2000 ms) appeared to allow updating and rehearsal processes (Bunting et al., 2006). In addition, Broadway and Engle (2010, p. 569) argued that “unless explicit instructions to rehearsals are given and enforced, it should not be assumed that participants engage in WM updating in running memory span”. Following this line of evidence, the present study used a fast presentation rate (500 ms) for the running span task, and we believe it mainly measures the sizes of FOA.

To sum up, although a neat one-to-one mapping relationship between WM tasks and WM functions is rare (if not impossible), the present study followed most studies in the literature (e.g., Morales et al., 2015; Dong and Liu, 2016) and used the n-back task to test our first hypothesis that the updating function of WM is closely related to CI performance, and CI training enhances updating. Similarly, we used the listening span task and a fast version of the letter running span task to test our second hypothesis that verbal WM spans are closely related to CI performance, and CI training enhances verbal WM spans. To increase data reliability and validity, we adopted a more-controlled design, and to reduce the influence of the complexity problem in interpreting training, we focused on CI and compared it with general L2 learning.

## Materials and Methods

### Participants

Two groups of young adult Chinese learners of English (93 in total, mean age = 19.72 years, SD = 0.79) were recruited at a university in China, and they received, respectively, two critical courses: general L2 training (EGP: English for General Purposes, 43 participants) and CI training (50 participants). All participants were non-English majors, and prior to the pretest, they had received no interpreting training. The two courses were optional but regular and registered courses in the university, and students were granted credits officially for the course they selected. The course of EGP was mainly an introduction to English culture and communication, with half class time spent on lecturing and the other half on student discussions (altogether 32 h of class time). Teachers and students were required to speak in English in the classroom. For the course of CI (mainly from L2 English to L1 Chinese), one third of the class time was spent on lecturing (e.g., illustrating interpreting strategies that mainly relates to effective source language comprehension, effective memory, and effective transmission) and two thirds on practice (altogether 32 h of class time). Apart from the 32 h of class time in each critical course, the two groups of participants did not differ either in their after-class practice (about 40 h in each course as indicated by data collected after the posttest). The two instructors for the two courses had been teaching their respective course for many years. Furthermore, the two courses were comparable in class size (50 students for each class).

Apart from the two critical courses, the participant groups were comparable in their trainings in other courses in the experimental semester. First, each group spent 32 h of class time (plus 56 after-class practice) in the course of “Comprehensive English” which includes trainings of basic skills of listening, speaking, reading, writing, and translation. Second, each group spent 256 h of class time in courses not related to language training (English).

### Procedure and Tasks

All participants were tested twice (pretest and posttest), respectively, at the beginning and end of an academic semester of 16 weeks. All participants took the pretest in the following order: a questionnaire, the L2 listening span task, the letter running span task, a cloze test, the n-back task, and an IQ test; and the posttest in the following order: a questionnaire, the three WM tasks, and CI test.

An English cloze test (Bachman, 1985) was used to test L2 proficiency (30 points in total),^3^ while Raven’s Advanced Progressive Matrices Set (Raven et al., 1977) to collect participants’ IQ (72 points in total). The composite questionnaire in the pretest was used to collect information about participants’ self-rated L2 proficiency, age, and parental education (Marian et al., 2007), and the questionnaire in the posttest was used to collect information about participants’ self-rated L2 proficiency and courses they took in the experimental semester. There were altogether 40 points in self-rated L2 proficiency, i.e., overall score of listening, speaking, reading, and writing, respectively, on a 10-point Likert scale. Father or mother education varied between 1 and 6, with 1–6, respectively, representing one’s highest diploma in primary school, middle school, high school, college (2-year professional training), university (4-year university education), and graduate education.

Three tasks were used to test participants’ WM: the visuo-spatial 2-back task, the L2 listening span task, and the letter running span task.

#### Visuo-Spatial 2-Back Task

Adapted from Soveri et al. (2011), the visuo-spatial 2-back version of the n-back task was used to measure participants’ updating function. After the instructions, a blue square was presented in one of 25 possible locations on the screen. Participants were asked to determine whether the location of the current square matched the location of the square before the previous one (2-back). There were altogether 42 2-back trials (28 non-target and 14 target trials). Each square remained on the screen for 500 ms and participants had to respond within 3000 ms. Participants were required to respond as quickly as possible without sacrificing accuracy. As indicators of participants’ updating ability, both RT and accuracy rate were computed.

#### L2 Listening Span Task

Adapted from Cai et al. (2015), the task consisted of 60 English sentences, each of which contained 8–12 words. Half of the 60 sentences did not make sense (e.g., “You’ve got to be more delicious about your future”). The non-sense version was created by simply replacing one or two words (e.g., “optimistic” with “delicious”) from an otherwise normal sentence. This way of creating the material followed the practice by Unsworth et al. (2009) (also see McVay and Kane, 2012; Redick et al., 2012, for examples). Participants were asked to listen through earphones to sentences auditorily presented and to remember the last word of each sentence. After hearing each sentence, participants were asked to make a judgment of the acceptability of the sentence by pressing one of the two keys. Sentences were presented in sets, and after the acceptability judgment of the last sentence in each set, participants heard an auditory cue (a “Ding” sound) and began to recall the last word of each sentence presented. No constraints were imposed on the order of recall except that participants should not begin with the last word of the last sentence in each set^4^. The set size (number of sentences) varied from two to six, with three trials in each set size. The presentation order of set size was randomly arranged for each participant. L2 listening span was the number of words correctly recalled (maximum score = 60).

#### Letter Running Span Task

Adapted from Broadway and Engle (2010), the task asked participants to recall the last *n* letters (targets) in the order of presentation from a list of *m* + *n* letters (inputs). There were a total of four sets of unrelated letters, with three lists of letters in each set. The number of letters to be recalled varied across sets (from 3 to 6) and was randomly ordered. Within each set, the input length was varied across lists, ranging from 3 to 8 letters. Each set began with instructions informing participants the number of last letters to be recalled, followed by the presentation of three lists of letters. Each list began with a fixation (“+”) for 500 ms, followed by visually presented letters one after another. Each letter was displayed for 300 ms and the interval between two letters was 200 ms. At the end of each list, participants recalled the letters they remembered by choosing among 12 letters displayed on the screen. A strict serial recall was required. No time constraint was set for recall. Before the experimental sequence, participants completed a practice set of four lists of unrelated letters, with the target length of two letters. Letter running span was the cumulative number of letters correctly recalled in lists where more letters were shown than were to be recalled (*m* > 0).

#### English–Chinese CI Test

At the end of the experimental semester, the CI group received an English–Chinese (E–C) CI test, in which participants were required to orally translate an English speech into Chinese. The test material is an approximately 6-min long coherent speech recorded by a native English speaker at an average rate of 143 words per minute. The speech was divided into segments, with each segment consisting of two to three sentences. Participants listened to each segment at a time, at the end of which a sound signal would indicate the time to start interpreting. The duration allowed for interpreting a segment was 1.5 times the duration of the segment itself. Another sound would then indicate the time to stop, and after a brief interval, a new segment was presented. Participants were allowed to take notes and refer back to these notes when they interpreted. Participants’ oral responses were recorded for scoring later. The CI materials (including the way of segmentation) had been used to assess students from the same population for many times before the present study, and both the students and teachers considered the materials appropriate for their CI assessment in terms of difficulty level, speed, and topic familiarity. Besides, no complaints about materials were made by our participants after the test. As for the scoring of participants’ interpreting products, we followed the criteria used before, which included information (accuracy and completeness, 70%) and target language (grammar and appropriateness, 30%).

## Results

### Data Trimming

Data from two participants (in the interpreting group) were excluded from further data analyses because of their abnormal performance in the IQ test (below 55 out of a total of 72, meaning “retarded” according to Raven et al., 1977). As described above, participants’ listening and letter running spans were, respectively, measured by the number of words or letters correctly recalled. Two measures were collected for the 2-back task: accuracy rate and RT. For n-back RT data, data from erroneous responses and those with RT less than 200 ms were discarded. Then outlier responses deviating by more than three SDs from the mean RTs for each participant were eliminated. Altogether less than 5% of extreme data (less than 200 ms, and outliers of three SDs from the mean) was affected (for the control group: pretest, 1.92%; posttest, 1.37%; for the interpreting group: pretest, 1.14%; posttest, 2.19%).

### Statistical Analyses

For clarity, the results of the statistical analyses are reported in two sections, answering two questions: How do CI training and general L2 training differ in their influences on WM? Are the updating and verbal span functions of WM related to CI performance?

#### How Do CI Training and General L2 Training Differ in Their Influences on WM?

To find out whether there were any group differences in the training effect, an analysis was conducted with Participant Group (CI group vs. control group) as the between-subject factor and Test Phase (pretest and posttest) as the within-subject factor. But before this analysis, we conducted a group comparison on the pretest measures to examine whether the two participant groups were comparable on the relevant factors that may influence WM performance or development. The result of this group comparison is shown in **Table 1**.

**Table 1:** Pretest group means (with SD) and comparisons of participants’ background characteristics and working memory (WM) task performances.

	Control (*n* = 43)	Interpreting (*n* = 48)	df	*t*-value	*p-*value
**Background characteristics**					
Interpreting	No	No			
Tested L2 proficiency	13.79 (3.55)	12.97 (3.61)	89	1.077	0.284
Self-rated L2 proficiency	19.67 (4.60)	20.10 (5.74)	89	-0.391	0.697
Age^∗^	19.86 (0.88)	19.58 (0.71)	89	1.652	0.102
AoA^∗^	9.02 (2.44)	9.29 (2.28)	89	-0.541	0.590
Father education^∗^	2.39 (0.69)	2.75 (1.26)	89	-1.632	0.106
Mother education^∗^	1.97 (1.01)	2.16 (1.21)	89	-0.808	0.421
Intelligence	67.51 (2.43)	66.56 (3.25)	89	1.561	0.122
**WM task performances**					
L2 listening span	26.20 (7.01)	26.10 (5.49)	89	0.080	0.936
Letter running span	23.60 (4.18)	22.38 (5.30)	89	1.218	0.226
2-back: RT	843.06 (273.11)	870.92 (265.92)	89	-0.493	0.623
2-back: accuracy^∗^	0.85 (0.095)	0.84 (0.087)	89	0.477	0.634

*^∗^Data for these variables were not normally distributed, and therefore Mann–Whitney tests were conducted, resulting in the same patterns as shown by independent oxt-test. For Age: U = 844.00, Z = -1.65, p = 0.098; for AoA: U = 986.50, Z = -0.37, p = 0.713; for Father education: U = 910.50, Z = -1.02, p = 0.306; For Mother education: U = 952.00, Z = -0.67, p = 0.506; For 2-back accuracy: U = 955.00, Z = -0.61, p = 0.540*.

The results of the comparisons, as revealed in **Table 1**, yielded no significant group differences in any of the indices of WM capacity or in L2 proficiency. *Since students selected their courses out of their own will, the null group differences suggest that students of interpreting did not choose the CI course because of some preexisting advantage in WM*.

**Table 2** presents participants’ posttest performance, and their gains from pretest to posttest, together with the group difference in each gain. The gains seem to indicate that the CI group tended to make more progress than the general L2 group in each of the four WM indices. However, a significant group difference was found only in updating RT (2-back RT) (*p* = 0.025) but not in the other three indices, which can also be shown by the effect size. According to a rough interpretation of the effect size values (Cohen, 1992), the group difference of updating RT (2-back RT) in gains from the pretest to the posttest was medium in effect size (*d* = 0.48), while that of letter running span was small (*d* = -0.22), and those of L2 listening span and updating accuracy were between small and medium (*d* = -0.35, -0.31).

**Table 2:** Posttest group means (with SD) in WM, their gains from pretest to posttest, and the group difference in each gain.

	Control group (*n* = 43)		Interpreting group (*n* = 48)		Group difference in each gain		
	Posttest mean	Gain	Posttest mean	Gain	*t-*value	*p*-value	Effect size *d*
L2 listening span	27.97 (7.68)	1.77 (5.37)	29.75 (6.29)	3.65 (5.49)	-1.645	0.103	-0.35
Letter running span	23.27 (5.19)	-0.33 (5.44)	23.20 (4.99)	0.82 (4.96)	-1.062	0.291	-0.22
2-back: RT	841.74 (261.17)	-1.32 (225.44)	766.15 (217.20)	-104.77 (206.17)	2.287	0.025	0.48
2-back: accuracy	0.88 (0.087)	0.03 (0.072)	0.90 (0.069)	0.06 (0.076)	-1.450	0.151	-0.31
CI performance			85.40 (6.21)				

Analysis of variance (ANOVA) was further conducted to find out how each group progressed in each index of data. **Table 3** shows the result of ANOVA (SPSS Statistics Version 19), with Participant Group as the between-subject factor (CI group, control group) and Test Phase as the within-subject factor (pretest, posttest) (see **Table 1** for pretest performance and **Table 2** for posttest performance).

**Table 3:** Summary of Participant Group × Test Phase analyses for each task index of the WM tasks.

	Main effect of phase			Main effect of group			Interaction effect		
	*F*(1,89)	*p*	$\eta_{p}^{2}$	*F*(1,89)	*p*	$\eta_{p}^{2}$	*F*(1,89)	*p*	$\eta_{p}^{2}$
L2 listening span	22.486	0.000	0.202	0.431	0.513	0.005	2.707	0.103	0.030
Letter running span	0.216	0.643	0.002	0.540	0.464	0.006	1.127	0.291	0.013
2-back: RT	5.497	0.021	0.058	0.243	0.623	0.003	5.228	0.025	0.055
2-back: accuracy	31.816	0.000	0.263	0.019	0.890	0.000	2.101	0.151	0.023

	**Simple effect (of progress) for participant groups**					
	**Control**			**Interpreting**		
	
2-back: RT	*p* = 0.968, *r* = 0.006			*p* = 0.001, *r* = 0.457		

As can be seen in **Table 3**^5^, the main effect of Test Phase (pretest vs. posttest) was significant in L2 listening span and the two indices of the 2-back task, reflecting a general training or practice effect. No main effect of Group was found in any index of the three WM tasks. The interaction effect was significant only in 2-back RT (*p* = 0.025, $\eta_{p}^{2}$ = 0.055). *The simple effect analysis showed that the interpreting group made a significant progress in 2-back RT (p = 0.001, r = 0.457), while the control did not make any progress (p = 0.968, r = 0.006)^6^*.

#### Are the Updating and Verbal Span Functions of WM Related to CI Performance?

We first conducted correlation analyses and the results are summarized in **Table 4**. As shown in **Table 4**, among the pretest WM indices, 2-back RT and listening span significantly correlated with CI performance, while for the posttest WM indices, only 2-back RT significantly correlated with CI performance.

**Table 4:** Correlations between CI performance and pretest or posttest WM

	2-back RT		2-back ACC		Letter span		Listening span	
	Pre-	Post-	Pre-	Post-	Pre-	Post-	Pre-	Post-
CI	-0.377^∗∗^	-0.294^∗^	0.091	-0.218	0.017	0.111	0.301^∗^	0.197

*^∗∗^, ^∗^Correlation significant, respectively, at the 0.01, 0.05 level (2-tailed)*.

Since both pretest 2-back RT and listening span significantly correlated with CI performance (*p* = 0.008 and *p* = 0.038, respectively), we further conducted a hierarchical multiple regression analysis to see whether both factors significantly predicted CI performance in the posttest (two predictors: pretest 2-back RT and listening span; one dependent variable: posttest CI performance). As seen in **Table 5**, pretest 2-back RT significantly predicted CI performance (*p <* = 0.012), while this prediction was only marginally significant for L2 listening span (0.05 < *p* < 0.10). The result of marginal significance for L2 listening span may seem strange because the correlation was significant (see **Table 4**), and the two pretest indexes of 2-back RT and L2 listening span did not correlate with each other for the interpreting group (*r* = 0.023, *p* = 0.541), showing the absence of multicollinearity. And yet, with the two variables entering the same regression model, the significance value did change (see **Table 5**). Since the correlation coefficient of 0.301 for L2 listening span was not very large (*p* = 0.038), it is understandable that the prediction was only marginally significant.

**Table 5:** Summary of hierarchical multiple regression analysis on the predictive effects of pretest WM (ID: independent variables) on CI performance (DV: dependent variable).

	DV	Block	IV	Δ*R*^2^	Δ*F*	Δ*p*	Beta
1	CI	1	2-back RT	0.142	7.436	0.009	-0.377
		2	Listening span	0.053	2.916	0.095	0.232
2	CI	1	Listening span	0.069	3.323	0.075	0.262
		2	2-back RT	0.126	6.909	0.012	-0.357

In short, among the three WM functions we have tested, only updating in the pretest significantly predicted CI performance in the posttest, and in the posttest, only updating significantly correlated with CI performance. Pretest L2 listening span correlated CI performance, but when entering the same regression model with updating, the prediction effect was only marginally significant.

## Discussion

With an intention to contribute to the issue of how language experience influences executive functions, the present study tried to reduce the problem of complexity in language experience, and focused on CI training and its effects on WM. With an analysis of the distinctive features of CI compared with general L2 learning in terms of WM requirements or involvement, we hypothesized that: (1) the updating function of WM was closely related to CI performance, and CI training could help enhance updating, (2) verbal WM spans may correlate with CI, and CI training may help enhance verbal WM spans. To increase the reliability of experimental data, we adopted a longitudinal design with a sufficient sample size and with participant groups that were controlled in their background characteristics (e.g., age, intelligence, social economic status, and language learning history). There were three main results: (1) Among all the WM indexes, only pretest and posttest n-back RT and pretest L2 listening span correlated with posttest CI performance; when both pretest n-back RT and pretest L2 listening span entered the same regression model, n-back RT significantly predicted posttest CI performance while this prediction was only marginally significant for L2 listening span. (2) For the index of n-back RT, the two participant groups differed significantly, with the interpreting group making a significant progress in the posttest, and the control group making no progress, while for the index of n-back accuracy, both groups improved equally significantly in the posttest. (3) For the two indices of WM spans, the two groups did not differ from each other, with both groups having improved significantly in L2 listening span in the posttest and neither group getting improved in the letter running span. In a word, our first hypothesis has been verified, and our second hypothesis was only weakly and partly supported.

### The Two Hypotheses on WM and CI

In our point of view, the most important finding for the present study is the relationship between updating and CI: updating efficiency (here specifically referring to updating speed based on a relatively high accuracy) predicted CI performance (see **Table 5**)^7^, and CI training enhanced updating efficiency (while general L2 training did not, see **Table 3**). Our analysis of the CI task in Section “Introduction” tried to explain this finding: recalling source language information in the target language is more demanding in terms of accuracy and speed when compared to listening and speaking in general L2 learning, and the process of recalling seems to match well with the process of updating as tested in the n-back task. For the prediction part of this finding (**Table 5**), we did not find similar reports in the literature, but we did replicate this prediction result in our later studies (with participants of higher L2 proficiency receiving much more CI training; manuscripts being prepared). The closest case in the literature was reported by Timarova et al. (2014) who found that professional interpreters of higher accuracy rates in a letter 2-back task performed better in the interpretation of numbers. For the enhancement part of this finding (**Table 3**), two previous studies reported similar results. Morales et al. (2015) reported better updating ability from simultaneous interpreters (SIs) when compared to general bilinguals. Dong and Liu (2016) found that CI training significantly enhanced updating ability, while the two control groups of written translation or general L2 training made only marginal or no progress. When comparing findings from the literature, we have to pay attention to the fact that professional interpreter or SIs are consecutive interpreters at the same time, but the other way around is not necessarily true.

The second hypothesis concerning the relationship between verbal WM spans and interpreting training was only weakly and partly supported. The pretest L2 listening span correlated with CI performance^8^, but when it entered the same regression model with the index of updating RT, the prediction effect was only marginally significant. In addition, there was a tendency for the interpreting group to make more progress in the posttest in both verbal span indices (a stronger tendency for L2 listening span than for letter running span, see **Table 2**), but that tendency was not significant (**Table 3**). These results are not good enough for us to make claims about the presence or absence of an interpreter advantage in WM spans. They could be reflections of the elusive nature of an interpreter advantage in complex verbal spans (generally verbal reading and listening spans) in the literature (e.g., advantage in Köpke and Nespoulous, 2006; Zhang, 2008; but no such advantage in Liu et al., 2004). With more training or with higher L2 proficiency, the relationship between CI performance and L2 listening span at least could be stronger, but more research is certainly needed to verify this hypothesis.

What we wish to emphasize is a potential connection between the two parts of the results for each hypothesis. (1) Results for Hypothesis One: updating efficiency predicted CI performance, and CI training enhanced updating efficiency (while general L2 training did not); (2) Results for Hypothesis Two: verbal WM spans did not significantly predict CI performance (i.e., only pretest L2 listening span correlated with CI performance and predicted CI performance with marginal significance), and CI training did not make a unique contribution to these spans. Briefly speaking, the reason for the second part probably lies in the first part. That is, the reason why one semester’s CI training brought participants an “interpreter advantage” in updating (but not in verbal spans) is probably that updating (but not verbal spans) is closely connected with the CI task, and therefore updating or a process parallel to updating (but not verbal spans) is trained in CI training. This process in CI is recalling information in the CI task. We may apply this line of thought to similar issues investigating how cognitive experience influences executive functions. Take the issue of “bilingual advantage” as an example. Experimental results for the bilingual advantage issue are quite mixed, most probably because inhibitory control is not more needed in some cases of bilingual experience than in monolingual experience, and therefore no inhibitory control advantage exists in such cases. Further research in this issue, therefore, may have to investigate the nature of bilingualism in terms of its involvement of executive functions when compared with monolingualism. For the present study, we may claim that CI involves updating more than it involves verbal WM spans. Whether this claim is true or false for similar cases of CI (e.g., CI by participants of higher L2 proficiency receiving more CI training) or for the relevant case of SI requires further research. More research of this line would fill blank patches in the dynamic picture for the relationship between interpreting and WM, which would then provide at least implications for the research on the relationship between cognitive experience and executive functions, and on the nature of cognitive experience itself.

Based on our theoretical analysis and experimental data, we believe that *updating and the recalling process in the CI task share the same attentional control process*. As analyzed in Section “Introduction,” the consecutive interpreter listens to a stretch of source language input, and then recalls as accurately as possible in the target language within a very short time. The Central Executive has to direct its attention backward to update information that has passed the FOA. This process is the same as the process in the n-back task in which the participant has to recall (or make judgments about) the stimulus that are n trials back. In other words, this updating attentional control process has been repeatedly exercised in CI training, leading to an interpreter advantage in updating.

### Methodological Issues

The experimental results in the present study also touched some methodological issues. The first one concerns the result that the L2 listening span was significantly enhanced in both groups while the letter running span did not improve in either group. This contrast is most probably a result of encoding more meaningful and less meaningful materials in the two tasks. Specifically, there are two possible reasons. First, the L2 listening span improved in both groups because both groups received language training in the pretest-posttest interval, while it may be too difficult for the language trainings to produce any effect on the letter running span in a short time. Second, since the materials of sentences in the L2 listening span task were meaningful, or more meaningful than the letters in the letter running span task, the meaningfulness may have produced more practice effect from the pretest itself, and may have benefited more from the trainings after the pretest. However, we believe that this difference between the two tasks did not affect much the major conclusions in the present study, because the design with a control group in the present study must have alleviated potential influences.

The second methodological issue concerns experimental control in the research on interpreter advantage. Although the problem of lack of control exists in the research on bilingual advantage, it seems more serious in the literature of research on interpreting, most probably because interpreters, especially simultaneous or professional interpreters, are not easily available. Nevertheless, rigid control is essential to valid conclusions no matter how hard it is to find matched groups of participants. Apart from what we have discussed about sample size in Section “Introduction,” participants need to be controlled in their *age*. Professional interpreters are generally older than students of interpreting, but WM capacity declines as a function of age (e.g., Charlton et al., 2010; Caplan et al., 2011). That could explain the finding that, in Signorelli et al. (2012), younger interpreters (mean age: 34.5) performed better than older interpreters (mean age: 56.2) in non-word repetition and cued recall tasks. Even among our participants that did not differ much from each other in age (19.86 years old with an SD of .88 for the control group), “age” negatively correlated with pretest letter running span (*r* = -0.255, *p* = 0.015) and posttest L2 listening span (*r* = -0.267, *p* = 0.010). The second factor that needs to be controlled is *L2 proficiency*, which has been found to play a role in WM capacity (e.g., Service et al., 2002). This role was evidenced by Tzou et al. (2012), where participants with higher L2 proficiency showed larger WM capacity than those with lower L2 proficiency. This relationship was supported by many pieces of evidence in the present study. For example, “tested L2 proficiency” correlated with pretest 2-back RT (*r* = 0.237, *p* = 0.024) and pretest L2 listening span (*r* = 0.274, *p* = 0.009). Besides these two factors, other factors like intelligence, socioeconomic status (SES) may also contribute to the differences and need to be controlled. For example, a significant unique variance in fluid intelligence was associated with WM (e.g., Shelton et al., 2010), and SES was found to explain a significant portion of the variance in cognitive achievement including WM (e.g., Noble et al., 2007). The present study found a marginally significant correlation between father education and L2 listening span (*r* = 0.199, *p* = 0.058).

Conducting longitudinal studies may help in experimental control, but there must be a control group matched with the experimental group in relevant background characteristics. Without the control group, the present study would have reached the probably wrong conclusion that interpreting training significantly enhanced L2 listening span (see the significant “main effect of testing phase” in **Table 3**). With the control group, we cannot reach that conclusion because of the null “interaction effect” in **Table 3**. This null effect indicates that the significant gain in L2 listening span in the posttest could be a result of the mixed effects of repeated testing (i.e., pretest and posttest), general L2 training or even exposure, and genuine influence of interpreting training. There are similar cases in the literature. For example, Zhang (2008) conducted a longitudinal study with three groups of participants: beginning interpreting trainees, advanced interpreting trainees, and professional interpreters (their age means were 23.4, 23.6, and 32.3, respectively), and comparing each group’s pretest and posttest scores (paired *t*-test), found that the beginning trainees’ reading span and advanced trainees’ coordinating ability were significantly improved by the 6 months’ experience of interpreting. Macnamara and Conway (2014) tested twice a group of 21 bimodal bilinguals with 2 years in between when participants received SI between American Sign Language (ASL) and English, and found that interpreting training enhanced the WM component processes of coordination and transformation (as tested by backward digit span and letter–number sequencing), but not processes of storage and processing (as tested by reading span and operation span).

In addition, conclusions on “advantage” research depend on how the supposedly advantageous group and a control group differ in the first place. If we had recruited a control group that did not receive the 32 (class) hours of general L2 training (but well matched otherwise) during the experimental semester, the interaction effect of L2 listening span between Participant Group and Testing Phase in **Table 3** may have reached significance, which may then lead to results similar to those for n-back RT (i.e., “L2 listening span was significantly enhanced by interpreting training”). Our conclusion about the question of how interpreting training influences WM, therefore, is based on a comparison between interpreting training and general L2 training, i.e., the additive effect of interpreting training above general L2 training.

The present study may help resolve controversies on the letter running task. For this purpose, we ran a correlation analysis on the four indices of WM, the results of which are listed in **Table 6**. On the one hand, there was no significant correlation between the two indices of the n-back task and the index of letter running in either the pretest or posttest, suggesting that the letter running task cannot be taken as a measurement of updating as suggested in some previous studies (e.g., Morris and Jones, 1990; Conway et al., 2005; Friedman et al., 2006). Since Bunting et al. (2006) found that only a slow pace of presentation of the letters (e.g., 2000 ms) appeared to allow updating and rehearsal processes to take place, the insignificant correlation between the two tasks is expected because the lapse between letters in the present research was normal or shorter, i.e., 500 ms. On the other hand, the two span tasks (L2 listening span and letter running span) correlated with each other in the posttest, which is consistent with what Broadway and Engle (2010) may have suggested since they found that the running span significantly correlated with the complex spans of reading (and listening and reading spans correlated with each other, see Cai and Dong, 2012). Taking all the three tasks into consideration, the correlations between them (**Table 6**) further specify the idea that the letter running span is more closely related to the verbal span of L2 listening than to updating ability measured in the n-back task.

**Table 6:** Correlations between WM indices in the pre- and post-tests (Pearson correlation, 2-tailed, 91 participants).

	2-back RT		2-back ACC		Letter span	
	Pre-	Post-	Pre-	Post-	Pre-	Post-
2-back ACC	0.185	0.308^∗∗^				
Letter span	0.113	0.140	-0.001	0.003		
Listening span	-0.105	0.028	0.102	0.131	0.129	0.333^∗∗^


*^∗∗^0.001 < p < 0.05*.

To sum up, the most critical finding for the present study is that both parts of our first hypothesis about updating were fully supported while neither part of our second hypothesis about verbal WM spans was (i.e., only pretest L2 listening span correlated with CI performance and predicted CI performance with marginal significance). This suggests that updating efficiency (even when measured by a non-verbal version of the n-back task as in the present study) is more central to the CI task than WM size, at least for beginning student interpreters like ours, and is therefore more exercised in CI training, leading to an interpreter advantage in updating efficiency. The underlying mechanism is that updating and the recalling process in CI share the same attentional control process.

## Ethics Statement

The present study was exempt from this requirement because we did not interfere with the classes that the participants received. We only collected cognitive data at the beginning and end of the semester, and all participants gave written informed consent for data collection in accordance with the Declaration of Helsinki.

## Author Contributions

YD had the idea and design, and wrote 90% of the paper. YL managed the experiments, analyzed the data, and helped with the writing. RC designed part of WM tasks and helped with the writing.

## Conflict of Interest Statement

The authors declare that the research was conducted in the absence of any commercial or financial relationships that could be construed as a potential conflict of interest.

## Footnotes

1 This is consistent with Rouder’s Rule of Thumb, i.e., if one runs with-subject designs in cognition and perception, one often gets high-powered experiments with 20–30 participants so long as they run about 100 trials per condition (http://jeffrouder.blogspot.be/2017/09/the-justification-of-sample-size.html).

2 There are other ways to test verbal WM spans: L1/L2 reading/listening/speaking span tasks. Many studies have indicated that these spans are closely related to each other. In addition, Cai and Dong (2012) found that three factors were in play in these spans (together with non-verbal digit and spatial spans), and their influences are hierarchical with different distinguishing power, from relatively strong (information type: verbal or non-verbal), to medium (encoding modality: listening, reading, or speaking), to relatively weak (encoding language: L1 or L2). To be more specific, two findings were relevant to the present study: (1) three pairs of spans were most closely clustered: L1/L2 listening spans; L1/L2 reading spans; L1/L2 speaking spans. (2) Listening and reading spans clustered on the next scale point, which then clustered with speaking spans. Since the verbal spans closely correlate with each other, since the listening span closely clusters with the classical reading span (Daneman and Carpenter, 1980), and since the L2 listening span has been tested in the literature of interpreters’ WM advantage, we decided to choose the L2 listening span as an appropriate measure of verbal spans. Future studies are encouraged to include complex WM tasks in the L1, such as L1 listening span.

3 In a cloze test, participants are required to fill in blanks in a passage with words appropriate for the context. In the following example, the answers for the two blanks are *kinds, types* or *examples* for the first, and *to* for the second. The science of automatic control depends on certain common principles by which an organism, machine, or system regulates itself. Many historical developments up to the present day have helped to identify these principles. *For hundreds of years there were many (1) ______ of automatic control systems, but no connections were recognized among them. A very early example was a device on windmills designed (2) ______ keep their sails facing into the wind…*

4 Researchers interested in the relationship between WM and interpreting frequently adopted free recall in reading and listening span tasks (e.g., Padilla, 1995; Christoffels et al., 2006; Signorelli, 2008). There are two motivations for this: (a) The very first developers of the reading span task accepted free recall themselves as they said “Because the test proved so difficult, the subject was given credit for any set for which he recalled all sentence final words, irrespective of the order of recall” (Daneman and Carpenter, 1980, p. 458); (b) Köpke and Signorelli (2012) argued that randomly determined order constraints were not very likely to be relevant to interpreting because word order in interpreting was determined by syntactic and semantic factors. Therefore, in spite of the popularity of serial recall for such WM tasks in experimental psychology, we adopted free recall for the listening span task in the present study so that we can compare our results with those of relevant previous studies. However, we do encourage further studies adopting serial recall, especially studies comparing free recall and serial recall.

5 We did not intend to collect RT data for the two span tasks because most previous research on the topic did not collect RT for WM spans. And yet, we “unintentionally” recorded participants’ RTs for the listening span task, i.e., the time it took each participant to judge if each presented sentence made sense or not. An analysis of these RT data indicated that the main effect of Participant Group (*p* = 0.342, $\eta_{p}^{2}$ = 0.010) or that of Testing Phase (*p* = 0.107, $\eta_{p}^{2}$ = 0.029) or their interaction (*p* = 0.949, $\eta_{p}^{2}$< 0.001) was not significant. Future studies are encouraged to collect and analyze RT data for WM span tasks.

6 We had deleted two participants’ data because of their abnormal performance in the IQ test. However, the results were more or less the same if these data had been included. With all participants’ data included, the interaction effect was not significant in L2 listening span (*p* = 0.139, $\eta_{p}^{2}$ = 0.024), letter running span (*p* = 0.318, $\eta_{p}^{2}$ = 0.011), and 2-back accuracy rate (*p* = 0.083, $\eta_{p}^{2}$ = 0.033), but significant in 2-back RT (*p* = 0.023, $\eta_{p}^{2}$ = 0.056), with the interpreting group making a significant progress (*p* = 0.001, *r* = 0.457) while the control group did not (*p* = 0.968, *r* = 0.006).

7 As to why the correlation with CI performance was better in the pretest than in the posttest in the present study, it was probably related to the high negative correlation between pretest 2-back RT and 2-back RT gain (from pretest to posttest): *r* = -0.530, *p* < 0.001. In other words, participants with relatively low pretest updating ability tended to improve more in the posttest, and participants with relatively high updating ability had less room to improve.

8 The reason for why L2 listening span correlated significantly with CI performance in the pretest but not in the posttest is probably the same as explained in Footnote 7. That is, participants with relatively low pretest L2 listening span tended to improve more in the posttest, and participants with relatively high L2 listening span had less room to improve, as shown in the negative correlation between pretest L2 listening span and its gain in the posttest: *r* = -0.290, *p* = 0.005.
